# Novel Imaging Probes: From Design to Applications

**DOI:** 10.3390/ph16101506

**Published:** 2023-10-23

**Authors:** Kuo-Ting Chen

**Affiliations:** Department of Chemistry, National Dong Hwa University, Hualien 974301, Taiwan; ktchen26@gms.ndhu.edu.tw

Molecular imaging has emerged as a powerful tool for clinical diagnosis. Several imaging modalities, including positron emission tomography (PET), single photon emission computed tomography (SPECT) and optical bioluminescence/fluorescence imaging, rely on the administration of molecular probes to acquire imaging signals. To be considered suitable for clinical applications, an ideal molecular imaging probe should demonstrate exceptional affinity, selectivity, stability and ability to be economically produced [1]. As it involves multi-disciplinary fields, developing imaging probes requires the collaborative efforts of experts in chemistry, biology, pharmaceuticals, radiochemistry and clinics. Recently, the advancement of medical knowledge and innovative chemical tools has driven the progress and new development of imaging probes. In this Special Issue, we collect four research papers and one review article describing inspiring chemical designs on imaging probes to enhance imaging quality or extend their utilities.

Bifunctional chelators (BFCs) serve as stable linkages between radionuclides and biovectors. The intelligent selection of a BFC is capable of labeling different radionuclides, which facilitates the simultaneous development of diagnostic and therapeutic radiopharmaceuticals on the same biovectors [2,3,4]. Murce et al. (contribution 1) reported the synthesis and radiolabeling of 3p-*C*-NETA-ePSMA-16 with diagnostic radionuclides ^111^In, Al^18^F and therapeutic radionuclides ^177^Lu, ^213^Bi to evaluate them as PSMA-targeting agents for radiotheranostics. Radiolabeling processes using the above radionuclides were efficient. The resulting two imaging probes, [^18^F]AlF-3p-*C*-NETA-ePSMA-16 and [^111^In]In-3p-*C*-NETA-ePSMA-16, both exhibited targeting specificity toward PSMA-positive tumors. Because of the rapid clearance of probes, short half-life radionuclides, such as ^213^Bi, were suggested for further therapeutic agent development based on 3p-*C*-NETA-ePSMA-16.

The use of click chemistry to develop imaging probes has emerged as a recent trend [5,6,7]. A bioorthogonal click reaction involving the condensation between *trans*-cyclooctenes (TCO) and tetrazine (Tz) in the inverse electron demand Diels–Alder (IEDDA) reaction is frequently used as a pre-targeting strategy [8,9,10]. Beaufrez et al. (contribution 2) employed a sultone ring-opening method to prepare hydrophilic [^18^F]fluorosulfotetrazine as a prosthetic agent, which can be further labeled with other biovectors via an IEDDA reaction. The new [^18^F]F-labeling agent exhibited fast clearance and no non-specific uptake in normal tissues, showing its potential for in vivo pre-targeting applications.

The fibroblast activation protein (FAP) is a “pan-tumoral” biotarget for molecular imaging and the treatment of various cancers [11]. Typical FAP-targeted ligands contain a 2-cyanopyrrolidine moiety, a glycine linker, and a quinolinyl group [12,13]. Based on these scaffolds, Lin’s group (contributions 3 and 4) reported two new series of FAP-targeted imaging probes in their in vivo studies. They found that the [^68^Ga]Ga-labeled pyridine-based FAP-targeted tracers revealed higher tumor-to-background ratios but lower tumor uptake compared to the standard probe. On the other hand, the pyrrolidinylboronic acid-based probes displayed low-to-medium tumor uptakes, though the replacement of the glycine linker with d-alanine enhanced tumor uptake. This research provides an in-depth structure–activity relationship (SAR) study for the development of FAP-targeted imaging probes.

A nanobody is the smallest antibody fragment comprising a single monomeric variable antigen-binding domain [14,15]. Bocancia-Mateescu et al. (contribution 5) reviewed recent developments in nanobody discovery, production and current challenges in applications for the diagnosis of cardiovascular diseases (CVDs). The authors also highlight the potential of nanobodies in the treatment of CVDs, including their ability to target specific biomarkers, act as labeling molecules, or assist in the delivery of drugs to specific targets. This review article provides a comprehensive overview of the current research on nanobodies and their potential applications in the field of CVDs, making it a valuable resource for researchers, clinicians and students interested in this topic.

In conclusion, this Special Issue underscores diverse and innovative approaches in developing imaging probes, ranging from bifunctional chelators for radiopharmaceuticals to click chemistry and nanobodies for targeted diagnostics and therapy. These advancements hold great promise for enhancing the field of molecular imaging and its applications in clinical diagnosis and treatment.

## List of Contributions

1. Murce, E.; Ahenkorah, S.; Beekman, S.; Handula, M.; Stuurman, D.; de Ridder, C.; Cleeren, F.; Seimbille, Y. Radiochemical and Biological Evaluation of 3p-*C*-NETA-ePSMA-16, a Promising PSMA-Targeting Agent for Radiotheranostics. *Pharmaceuticals* **2023**, *16*, 882.
2. Beaufrez, J.; Guillouet, S.; Seimbille, Y.; Perrio, C. Synthesis, Fluorine-18 Radiolabeling, and In Vivo PET Imaging of a Hydrophilic Fluorosulfotetrazine. *Pharmaceuticals* **2023**, *16*, 636.
3. Bendre, S.; Kuo, H.-T.; Merkens, H.; Zhang, Z.; Wong, A.A.W.L.; Bénard, F.; Lin, K.-S. Synthesis and Preclinical Evaluation of Novel ^68^Ga-Labeled (*R*)-Pyrrolidin-2-yl-boronic Acid-Based PET Tracers for Fibroblast Activation Protein-Targeted Cancer Imaging. *Pharmaceuticals* **2023**, *16*, 798.
4. Verena, A.; Kuo, H.-T.; Merkens, H.; Zeisler, J.; Bendre, S.; Wong, A.A.W.L.; Bénard, F.; Lin, K.-S. Novel ^68^Ga-Labeled Pyridine-Based Fibroblast Activation Protein-Targeted Tracers with High Tumor-to-Background Contrast. *Pharmaceuticals* **2023**, *16*, 449.
5. Bocancia-Mateescu, L.-A.; Stan, D.; Mirica, A.-C.; Ghita, M.G.; Stan, D.; Ruta, L.L. Nanobodies as Diagnostic and Therapeutic Tools for Cardiovascular Diseases (CVDs). *Pharmaceuticals* **2023**, *16*, 863.
